# Rapid recovery of male cats with postrenal acute kidney injury by treating with allogeneic adipose mesenchymal stem cell-derived extracellular vesicles

**DOI:** 10.1186/s13287-022-03039-z

**Published:** 2022-07-28

**Authors:** Weihui Li, Wei Wang, Xin He, Zheng Liao, Aili Aierken, Jinlian Hua, Yan Wang, Dezhang Lu, Shiqiang Zhang

**Affiliations:** 1 College of Veterinary Medicine, Shaanxi Center of Stem Cells Engineering and Technology, Northwest A&F University, Yangling, 712100 China; 2 Xi’an Animal Hospital, Northwest A&F University, Xi’an, China

**Keywords:** Acute kidney injury, Adipose mesenchymal stem cells, Extracellular vesicles, Cats

## Abstract

**Background:**

Acute kidney injury (AKI) is a complex disease and can be generally divided into prerenal, intrarenal, and postrenal AKI (PR-AKI). Previous studies have shown that mesenchymal stem cells (MSCs)-derived extracellular vesicles have protective function on prerenal and intrarenal AKI treatment, but whether they have therapeutic efficacy on PR-AKI remains unclear. In this study, we investigated the therapeutic efficacy of allogeneic adipose mesenchymal stem cell-derived extracellular vesicles (ADMSCEVs) on cat models of PR-AKI.

**Methods:**

The cat models of PR-AKI were established by using artificial urinary occlusion and then treated with ADMSCEVs. Histopathological section analysis, blood routine analysis, plasma biochemical test, imaging analysis, and plasma ultra-high performance liquid chromatography-MS/MS (UHPLC-MS/MS) were performed to evaluate the therapeutic efficacy of ADMSCEVs.

**Results:**

Physiological and biochemical test showed that the ADMSCEVs could recover creatinine, urea nitrogen and plasma phosphorus to homeostasis efficiently. Blood routine analysis showed that leukocytes in PR-AKI cats with ADMSCEVs treatment returned to normal physiological range more quickly than that of control. UHPLC-MS/MS analysis revealed that the plasma metabolome profile of PR-AKI cats treated with ADMSCEVs was highly similar to that of normal cats. Furthermore, UHPLC-MS/MS analysis also revealed six metabolites (carnitine, melibiose, d-Glucosamine, cytidine, dihydroorotic acid, stachyose) in plasma were highly correlated with the dynamic process of PR-AKI on cats.

**Conclusions:**

We demonstrate the efficacy of ADMSCEVs in the treatment of PR-AKI on cats. Our study also suggests six metabolites to be novel PR-AKI markers and to be potential targets for ADMSCEVs therapy. Our findings will be useful to improve clinical treatment of both animal and human PR-AKI patients with ADMSCEVs in the future.

**Supplementary Information:**

The online version contains supplementary material available at 10.1186/s13287-022-03039-z.

## Introduction

Acute kidney injury (AKI), also called acute renal failure, is a serious, rapid and complex disorder of kidney damage in human. The incidence rate of AKI is between 1/10000 and 6/10000 per year in the community setting [1]. According to the etiology, AKI can be generally divided into prerenal, intrarenal, and postrenal (PR-AKI). The main causes of prerenal AKI include decreased blood volume (such as massive hemorrhage and severe dehydration), heart failure (insufficient renal perfusion caused by decreased cardiac output), shock or the use of high-dose vasodilators. Intrarenal AKI occurs when direct damage to the kidneys, and is common in glomerular disease, tubulointerstitial disease, and nephrovascular disease. By contrast, PR-AKI occurs when an obstruction in the urinary tract causes the waste to build up in the kidneys. PR-AKI accounts for about 10% of AKI, especially in elderly patients, the proportion of PR-AKI is up to 22% [2].

Male cats are ideal models to study PR-AKI, since male cats have a long and narrow urethra, which is easy to induce urinary obstruction due to stress. The clinical symptoms of PR-AKI on cats are highly similar to those of humans. In clinical veterinary medicine, the proportion of PR-AKI in male cats is as high as 65%. Mortality for PR-AKI in cats is also high, approaching 47%. Nearly 60% male cats developed chronic renal failure of the survived individuals [3].

In mice and rats, previous studies have shown that mesenchymal stem cell-derived extracellular vesicles can effectively treat prerenal and intrarenal AKI [4–7]. So far, it is not clear whether mesenchymal stem cell-derived extracellular vesicles are also effective in the treatment of PR-AKI. In this study, we investigated the efficacy of allogenic adipose mesenchymal stem cell-derived extracellular vesicles (ADMSCEVs) on the cat models of PR-AKI. Through physiological and biochemical analysis, blood routine analysis and plasma metabolomic analysis, we found that ADMSCEVs therapy promoted the rapid recovery of cats with PR-AKI.

Kidney is an important regulatory organ of systemic metabolic homeostasis and has a decisive impact on plasma composition. However, it is unclear which components of plasma are regulated by ADMSCEVs in the treatment of PR-AKI. Ultra-high-performance liquid chromatography-MS/MS (UHPLC-MS/MS) is a powerful method for estimation of metabolites in blood. Using UHPLC-MS/MS, we further revealed six metabolites in plasma were highly correlated with the dynamic process of PR-AKI on cats and were potential novel targets for ADMSCEVs therapy. Our study provides valuable references and clues for translational medicine research on the treatment of human PR-AKI with ADMSCEVs in the future.

## Materials and methods

### Animals, isolation and culture of ADMSCs

The animal study was reviewed and approved by the Institutional Animal Care and Use Committee at Northwest A&F University (NWAFU.No20200620c0600025[176]). Healthy female cats (three months old) were used for isolation of adipose mesenchymal stem cells. After anesthesia, the groin adipose tissue was taken aseptically and cut into one mm^3^ pieces for collagenase digestion. Collagenase (type 1, Gibco) (0.1%) was added to the tissue pieces and incubated at 37 °C for one hour. Then, the supernatants were discarded by centrifuging at 250 g for 5 min. The cell pellets were resuspended with 5 ml MEM basal medium and filtered through a cell strainer. Finally, the cell pellets were collected by centrifugation (250 g, 5 min) again and cultured with ADMSCs medium (88% MEM basal medium (Gibco), 1% penicillin and streptomycin (Gibco), 1% glutamine (Gibco) and 10% fetal bovine serum (Biological Industries)). After 24 h, non-adherent cells were removed, and the cell culture was replaced with the fresh ADMSCs medium. When the ADMSCs grow up to 80% confluence, cells were trypsinized and collected for liquid nitrogen cryopreservation. In this study, ADMSCs within four passages were used for subsequent treatment trials.

### Differentiation of ADMSCs and characterization

Osteogenic differentiation: The ADMSCs at passage 4 were cultured in a 12-well plate with ADMSCs medium. When the cells growth reached 60% confluence, the ADMSCs were replaced with osteogenic differentiation medium (MEM medium containing 10% FBS, 100 nM dexamethasone (Selleck), 30 μg/ml ascorbic acid (sigma) and 10 mM sodium ß-glycerophosphate (Solarbio)) and differentiated for another 14 days. After that, the cells were fixed with 4% paraformaldehyde, washed with PBS, and incubated with alizarin red staining solution for 30 min. Finally, cells are used for photography after removing alizarin red staining solution and washing with PBS for three times.

Adipogenic differentiation: The ADMSCs were cultured similarly as above, then replaced with adipogenic differentiation medium (MEM medium containing 10% FBS, 5% Insulin-Transferrin-Selenium-Ethanolamine (ITS-X) (Selleck), 1 μM dexamethasone (Selleck) and 0.5 mM 3-Isobutyl-1-methylxanthine (IBMX) (Selleck)). After 14 days’ differentiation, the cells were fixed with 4% paraformaldehyde, washed with PBS, and incubated with oil red O staining solution (Solarbio) for 60 min. Finally, cells are used for photography after removing staining solution and washing with PBS for three times.

### Isolation and identification of ADMSCs-derived extracellular vesicles

The ADMSCs within the fourth passage in vitro were used for isolating extracellular vesicles. When the cells grew to 90% confluence, they were cultured in UltraGRO™-PURE (Helios) for 24 h and cell supernatants were collected for extracellular vesicles extraction with the TransExo® Cell Media Exosome Kit (TransGen biotech). Although the manufacturer claimed that TransExo® Cell Media Exosome Kit was designed to extract exosomes, we found that the extracted components included microvesicles (100–1000 nm) in addition to exosomes (30–100 nm). For more accuracy, we defined the extract as extracellular vesicles.

To characterize the ADMSCs-derived extracellular vesicles (ADMSCEVs), transmission electron microscopy was used for morphological examination. The size distribution and concentration of the isolated ADMSCEVs were observed by using Malvern Zetasizer Nano-ZS ZEN 3600. The ADMSCEVs pellets lysate was immunoblotted for TSG101 (1:1500, Wanleibio) and CD63 (1:1000, Wanleibio).

### Histopathological section analyses

Kidneys were surgically dissected from the experimental cats. Samples were fixed in PBS containing 4% formaldehyde, and embedded in paraffin. Sections were prepared in Xi 'an Animal Hospital of Northwest A&F University, and stained with hematoxylin and eosin.

### Cat models of PR-AKI and treatments

Healthy male cats (body weight, 2.5–3.5 kg) were raised in a quiet environment for one week to reduce stress response before surgery. To establish a model of artificial PR-AKI, venous access was established first, and then propofol anesthesia was performed. After anesthesia, the cat's penis was shaven and disinfected with iodophor. The catheter with a diameter of 1.3 mm was inserted completely into the urethra. After that, the catheter was closed with heparin cap. Furthermore, the catheter was sutured to the foreskin to prevent it from falling off. The plasma of experimental cats was collected and tested by Fuji biochemical analyzer, and the blood routine was determined by Mindray four classification hematology analyzer. The cats with plasma creatinine value exceeded 770 μM were identified as a successful model of artificial PR-AKI and used for subsequent treatment trials.

For treatment trials, PR-AKI cats were randomly divided into control treatment group (*n* = 3) and ADMSCEVs treatment group (*n* = 3). The treatment procedure was as follows: On the first day of treatment, all the PR-AKI cats were relieved from urinary closure. Meanwhile, the control group and ADMSCEVs group were treated with intravenous infusion of different components. The control treatment group was infused with ceftiofuroxime sodium (5 mg/kg), 5% glucose, and 0.9% sodium chloride. The ADMSCEVs treatment group was infused with extracellular vesicle extract (the infusion dose was equivalent to the amount of extracellular vesicles secreted by 10 million cat ADMSCs in 24 h), plus ceftiofuroxime sodium (5 mg/kg), 5% glucose, and 0.9% sodium chloride. Extracellular vesicle extract was used only once on the first day of infusion. On the second and third day of treatment, both groups received the same infusion components (ceftiofuroxime sodium (5 mg/kg), 5% glucose, and 0.9% sodium chloride). The total amount of infusion liquid was calculated as 50 mL/kg weight + daily urine volume (mL), and the infusion speed was 10 mL/kg/h. During the treatment period, venous blood was collected every day for further analyses.

### UHPLC-MS/MS analyses

Plasma samples of cats were thawed at 4 °C, and 100 μL was taken from each sample, and mixed with 100 μL of pre-cooled ultrapure water and 800μL of pre-cooled methanol/acetonitrile (1:1, V/V). Ultrasound was performed in the ice bath for 60 min, then the mixture was then incubated at − 20 °C for 2 h, and centrifuged at 16,000*g* at 4 °C for 30 min. The supernatants were evaporated in a high-speed vacuum concentration centrifuge. The pellets were re-dissolved with 100 μL acetonitrile–water solution (1:1, v/v), centrifuged at 16,000*g* for 20 min at 4 °C, and the supernatant was taken for UHPLC-MS/MS analysis.

During the whole UHPLC-MS/MS analysis, the samples were placed in an automatic sampler at 4 °C, metabolomics profiling was analyzed using a UPLC-ESI-Q-Orbitrap-MS system (UHPLC, Shimadzu Nexera X2 LC-30AD, Shimadzu, Japan) coupled with Q-Exactive Plus (Thermo Scientific, San Jose, USA). Injection volume was 3 μL, the column temperature was 25 °C, and the flow rate was 0.3 mL/min. Chromatographic mobile phase A: water plus 25 mM ammonium acetate; B: 100% acetonitrile. The chromatographic gradient elution procedure was as follows: the gradient was 95% B for 1 min, and linearly reduced to 65% in 7 min, and then reduced to 35% in 2 min and maintained for 1 min, and then increased to 95% in 0.5 min, with 2 min re-equilibration period employed.

### Mass spectrum acquisition

The positive (+) and negative (−) modes of each sample were detected by electrospray ionization (ESI). Samples were separated by UPLC and analyzed by MASS spectrometry using QE Plus Mass Spectrometer (Thermo Scientific). HESI source conditions were set as follows: Spray Voltage: 3.8 kv (+) and 3.2 kv(−); Capillary Temperature: 320 (±); Sheath Gas: 30 (±); Aux Gas: 5 (±); Probe Heater Temp: 350 (±); S-Lens RF Level: 50.

Mass spectrometry collection time was 12 min. Parent ion scanning range: 80–1200 m/z, primary mass spectrometry resolution: 70,000@m/z 200, AGC target: 3e6, primary maximum injection time: 100 ms. The secondary mass spectrum analysis was collected according to the following methods: isolation window was set to 2 m/z, maximum injection time: 50 ms, and the normalized collision energy (stepped) was set at 27, 29 and 32 for fragmentation.

Quality control (QC) samples were prepared by pooling aliquots of all samples that were representative of the samples under analysis, and used for data normalization.

### Metabolomics data preprocessing, filtering and analysis

MSDIAL software was used for peak alignment, retention time correction and peak area extraction for the raw data. The metabolites were identified by measuring accurate  mass (mass tolerance < 0.01 Da) and MS/MS data (mass tolerance < 0.02 Da) were matched with HMDB, MassBank, and self-built metabolite standard library which was generated by Shanghai Bioprofile Biological Technology Co., LTD.

In the extracted-ion features, only the variables having more than 50% of the nonzero measurement values in at least one group were kept. The positive and negative ion peaks were integrated and R software was used for pattern recognition. Further data analysis, including principal component analysis (PCA), differential metabolite analysis, and KEGG pathway screening, were performed by Metaboanalysis 5.0. The rest of data was analyzed by using GraphPad Prism 6.0.

## Results

### Urethral obstruction in male cats mimics the clinical symptoms of PR-AKI

**Fig. 1:**
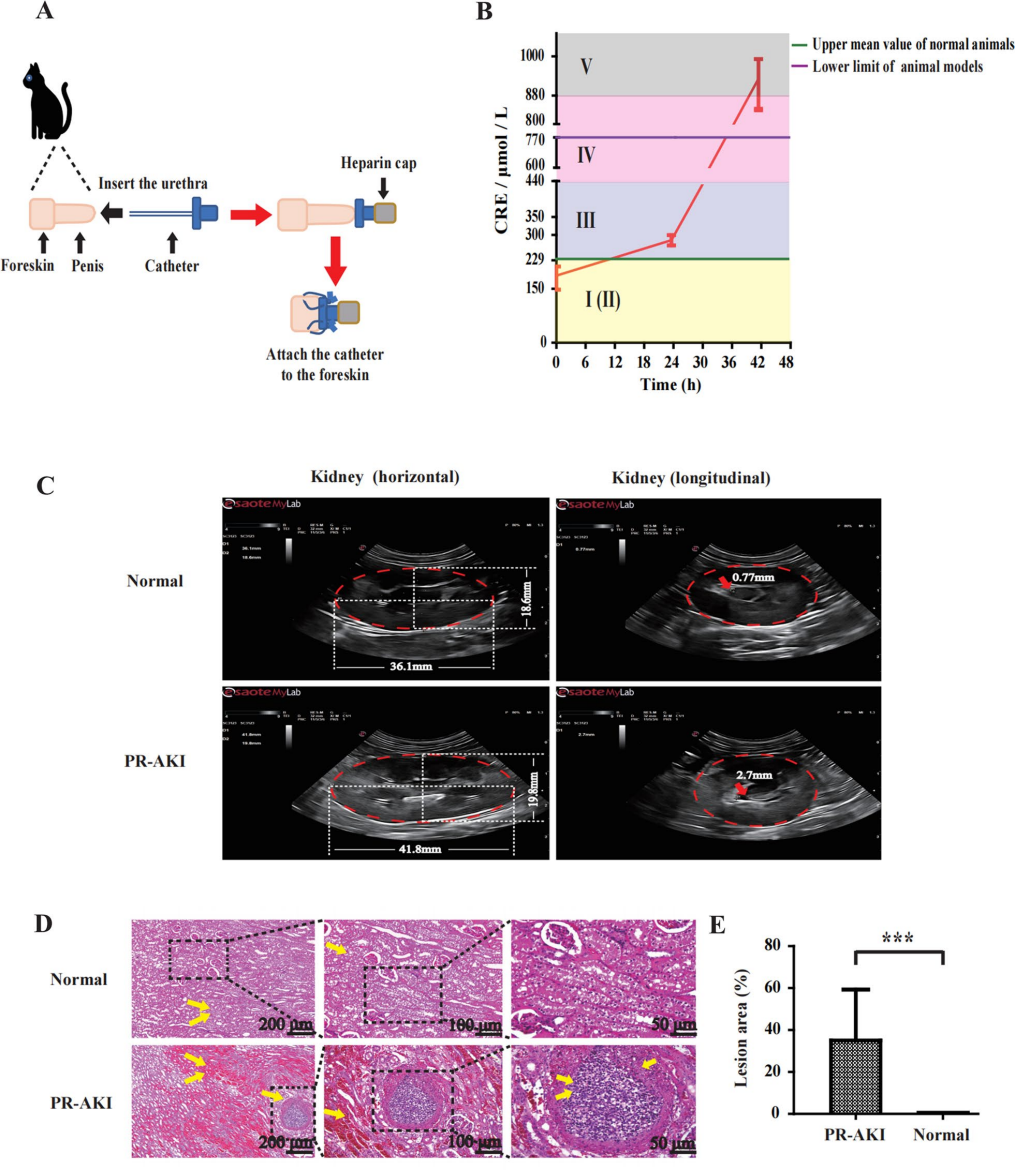
Male cat model of artificial urethral blockage mimics the clinical symptoms of PR-AKI. **A** Schematic diagram of a male cat model of artificial urethral blockage. **B** Dynamic changes on creatinine (CRE) levels at different time points of artificial urethral blockage in male cats. **C** B-ultrasound imaging of kidneys in male cats with artificial urethral obstruction for 48 h. **D** HE staining of the kidney sections in male cats with artificial urethral obstruction for 48 h. **E** Proportions of lesion area in pathological sections, statistical analyses were performed using two-tailed unpaired *t* test, ***P < 0.001

To make a PR-AKI cat model, we inserted the catheter into the urethra of a male cat and closed the heparin cap for artificial urinary closure (AUC) (Fig. 1A). Creatinine levels in the blood were monitored for 48 h. AKI was classified into five grades based on creatinine levels proposed by International Renal Interest Society. About nine hours after AUC, the blood creatinine level increased to grade III AKI. About 30 h after AUC, the blood creatinine level increased to grade IV AKI. In this study, the blood creatinine level of 770 μM was set as the lower limit standard of PR-AKI cat models, which took about 35 h after AUC (Fig. 1B). B ultrasonic examination showed that the kidneys and ureters of PR-AKI cats both enlarged when compared to that of cats at the normal state (Fig. 1C). Furthermore, the HE staining of kidney sections of cats with PR-AKI showed obvious lesions (Fig. 1D, E).

### Therapeutic efficacy of ADMSCEVs on PR-AKI cats

**Fig. 2:**
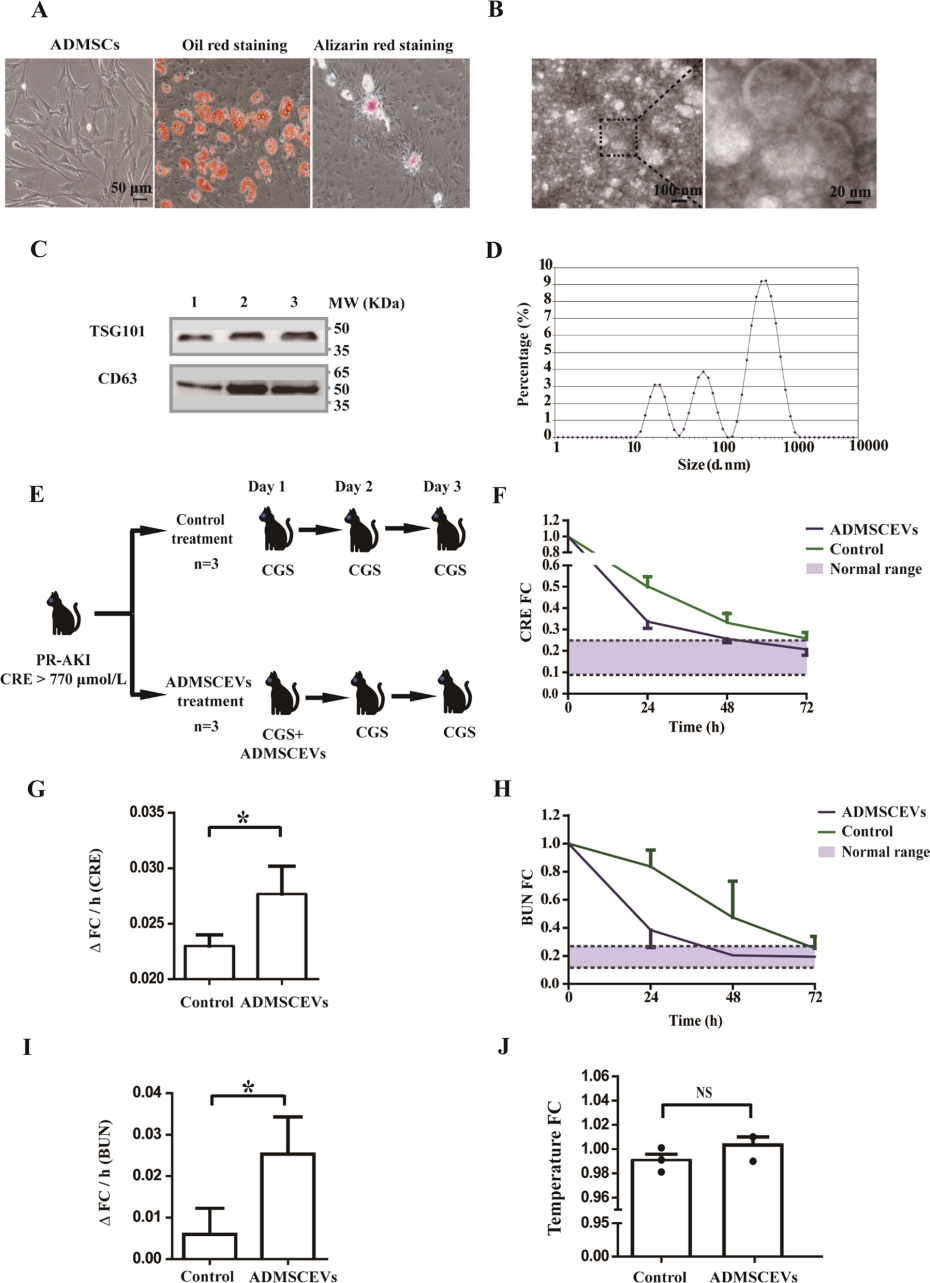
Treatment of PR-AKI cats by using the infusion with ADMSCEVs. **A** ADMSCs morphology and its differentiation into adipose and cartilage defined by oil red staining and alizarin red staining, respectively. **B** Transmission electron microscopy images of ADMSCEVs. Left panel, typical vesicle structure of ADMSCEVs, right panel, the enlarged ADMSCEVs. **C** Western blotting of ADMSCEVs marker protein TSG101 and CD63. The number 1, 2, 3 indicate three independent samples of ADMSCEVs. MW, molecular weight. **D** Particle sizes distribution of ADMSCEVs. **E** Schematic diagram of different treatment procedures for PR-AKI cats. CGS: Ceftiofuroxime sodium (C); Glucose (G); Sodium chloride (S). **F** Dynamic fold changes in creatinine level of PR-AKI cats treated by control infusion and ADMSCEVs. CRE FC, creatinine fold changes. **G** Average folds changes of creatinine level per hour in the first 24 h of treatment with the control infusion and ADMSCEVs. Statistical analyses were performed using two-tailed unpaired *t* test, *P < 0.05. **H** Dynamic fold changes in blood urea nitrogen level of PR-AKI cats treated by control infusion and ADMSCEVs. BUN FC, blood urea nitrogen fold changes. **I** Average folds changes of blood urea nitrogen (BUN) level per hour in the first 24 h of treatment with the control infusion and ADMSCEVs. Statistical analyses were performed using two-tailed unpaired *t* test, *P < 0.05. **J** Fold changes in body temperature of PR-AKI cats treated with control infusion and ADMSCEVs. *NS* not significant

To investigate the therapeutic efficacy of ADMSCEVs on PR-AKI cats, we first carried out quality control and identification of ADMSCEVs. The isolated ADMSCs had a typical mesenchymal-like morphology and could differentiate into adipocytes and osteoblasts (Fig. 2A). ADMSCs with the above differentiation potential were used for subsequent extracellular vesicles isolation. Transmission electron micrograph results showed that the extracted ADMSCEVs had typical vesicle structures (Fig. 2B). Western blotting showed that ADMSCEVs expressed the marker proteins TSG101a and CD63 (Fig. 2C). The size of the ADMSCEVs was measured, and the diameter of ADMSCEVs was mainly distributed around 620.9 nm, followed by 94.58 nm and 23.57 nm (Fig. 2D).

The whole process of treatments is shown in Fig. 2E. During the whole process of ADMSCEVs treatment, we monitored the dynamic parameters of blood routine and blood biochemistry of PR-AKI cats. PR-AKI cats with ADMSCEVs treatment returned to normal physiological levels more quickly than that with control treatment within 72 h in terms of creatinine and urea nitrogen (Fig. 2F, H). Notably, fold changes per hour of creatinine and urea nitrogen of PR-AKI cats treated with ADMSCEVs were significantly faster than that of control in the first 24 h (*P* < 0.05) (Fig. 2G, I). We found no significant differences in leukocytes, plasma phosphorus, and plasma calcium between ADMSCEVs and control treatment. However, ADMSCEVs treatment made PR-AKI cats recover faster in the above three indicators when compared with the control treatment (Additional file 2: Fig. S1A–C). In clinical manifestations, there was no significant difference in body temperature between ADMSCEVs and control treatment (Fig. 2J), indicating the safety of ADMSCEVs. In short, ADMSCEVs therapy can significantly promote the recovery of PR-AKI cats.

### Plasma metabolomic analysis of PR-AKI cats treated by ADMSCEVs

To comprehensively evaluate the efficacy of ADMSCEVs on PR-AKI cats, we performed a paired plasma metabolomic analysis of cats in normal physiology, PR-AKI, and ADMSCEVs treatment. We identified 10,145 positive ion features and 6607 negative ion features by using UHPLC-MS/MS. Furthermore, we got 460 annotated chemicals from 10,145 positive ion features and mapped these 460 chemicals on 192 KEGG pathways. In parallel, we got 348 annotated chemicals from 6607 negative ion features and mapped these 348 chemicals on 162 KEGG pathways (Additional file 1: Table S1).

**Fig. 3:**
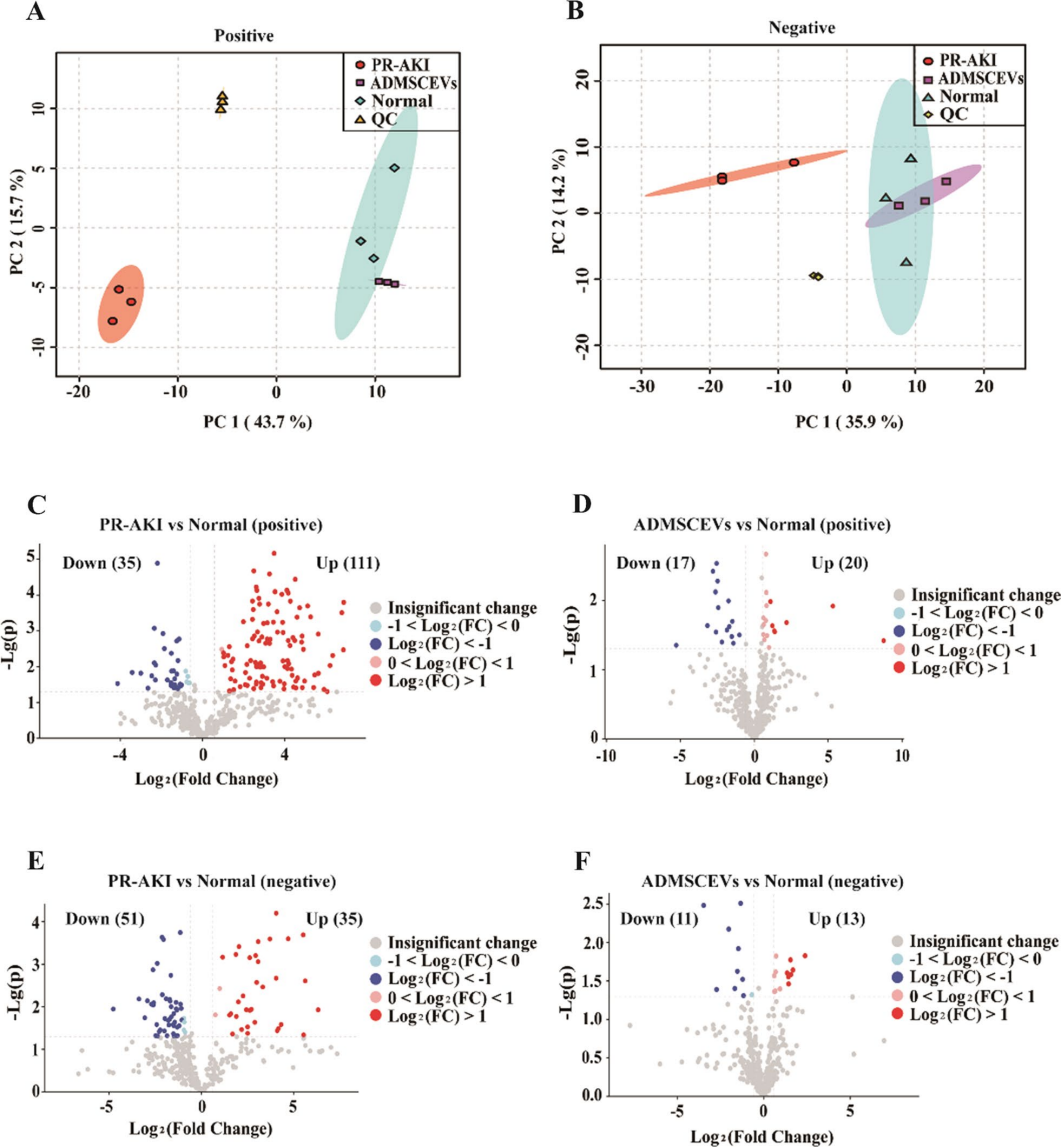
Plasma metabonomics analyses of PR-AKI cats treated with ADMSCEVs. **A**, **B** Principal component analysis of the positive ion features (**A**) and negative ion features (**B**). *QC* quality control. **C** Volcano diagram of differential metabolites (annotated from positive ion features) between PR-AKI cats and normal cats. **D** Volcano diagram of differential metabolites (annotated from positive ion features) between ADMSCEVs-treated cats and the normal cats. **E** Volcano diagram of differential metabolites (annotated from negative ion features) between PR-AKI cats and normal cats. **F** Volcano diagram of differential metabolites (annotated from negative ion features) between ADMSCEVs-treated cats and the normal cats

Principal component analysis of both positive ion features and negative ion features showed that the ADMSCEVs treatment group and the normal physiological group were clustered together, while the PR-AKI group was separated alone (Fig. 3A, B). In terms of annotated chemicals from positive ion features, PR-AKI group caused upregulation of 111 chemicals and downregulation of 35 chemicals compared with normal physiological group (*P* < 0.05, VIP > 1, Fold Change > 1.5, the same criteria was applied thereafter) (Fig. 3C). By contrast, ADMSCEVs treatment group caused only upregulation of 20 chemicals and downregulation of 17 chemicals compared with normal physiological group (Fig. 3D). In terms of annotated chemicals from negative ion features, PR-AKI group caused upregulation of 35 chemicals and downregulation of 51 chemicals compared with normal physiological group (Fig. 3E). In sharp contrast, ADMSCEVs treatment group caused only upregulation of 13 chemicals and downregulation of 11 chemicals compared with normal physiological group (Fig. 3F). In short, these results suggest that ADMSCEVs treatment effectively restored the plasma metabolome of PR-AKI cats to a level highly similar to normal physiological homeostasis.

### Potential chemical targets of ADMSCEVs therapy for PR-AKI

**Fig. 4:**
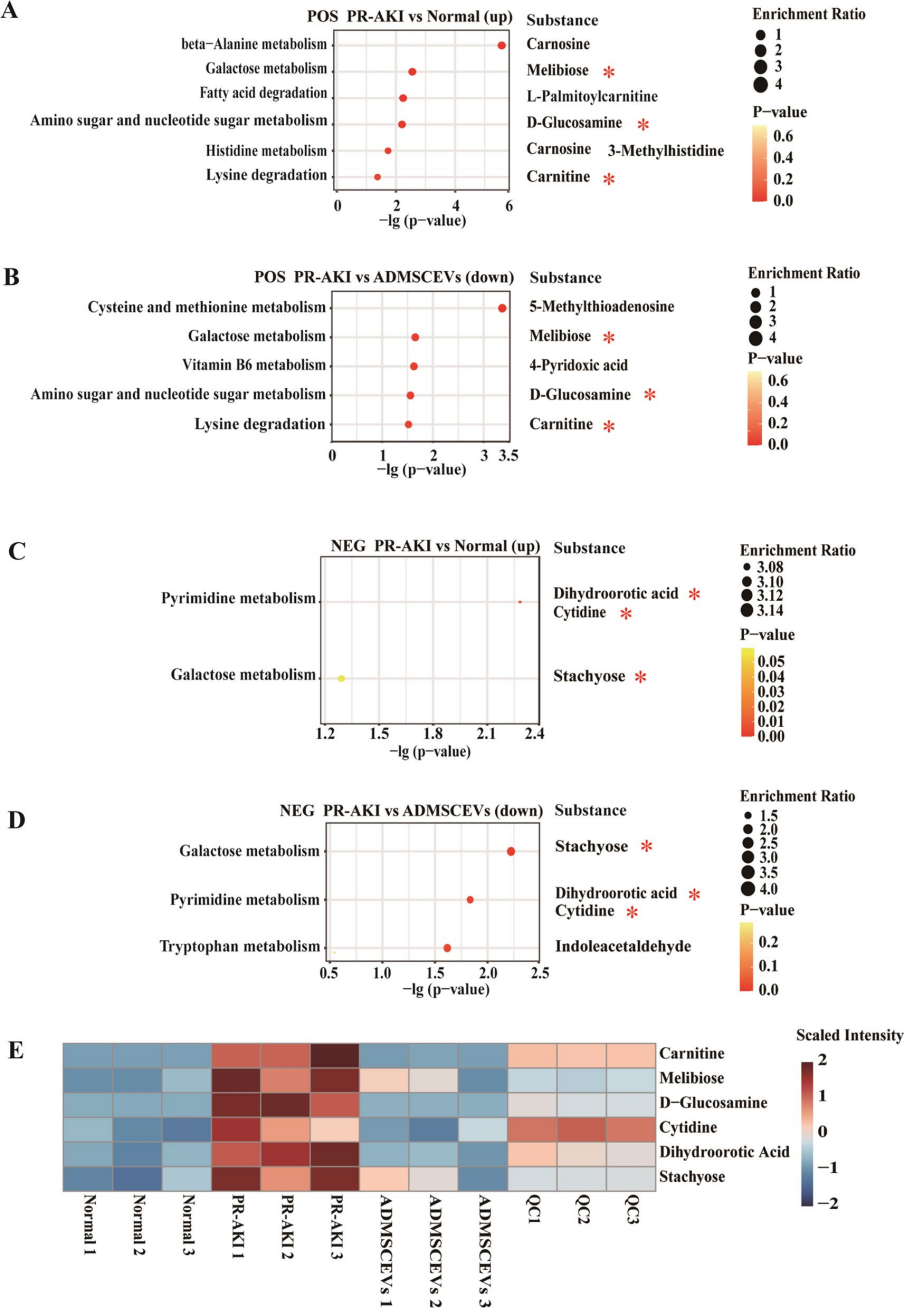
Potential targets of ADMSCEVs in the treatment of PR-AKI. **A** KEGG pathway analysis of significantly upregulated metabolites (annotated from positive ion features) in PR-AKI cats compared to normal cats. The metabolites enriched in the pathways with significant difference are listed on the right. *P* value < 0.05. **B** KEGG pathway analysis of significantly downregulated metabolites (annotated from positive ion features) in ADMSCEVs-treated cats compared to PR-AKI cats. The metabolites enriched in the pathways with significant difference are listed on the right. *P* value < 0.05. Common metabolites enriched in upregulated (**A**) and downregulated (**B**) pathways are marked with an asterisk. **C** KEGG pathway analysis of significantly upregulated metabolites (annotated from negative ion features) in PR-AKI cats compared to normal cats. **D** KEGG pathway analysis of significantly downregulated metabolites (annotated from negative ion features) in ADMSCEVs-treated cats compared to PR-AKI cats. Common metabolites enriched in upregulated (**C**) and downregulated (**D**) pathways are marked with an asterisk. **E** Heatmap showing the expression pattern of six metabolites as potential targets for ADMSCEVs treatment of PR-AKI

Then, we explored potential chemical targets of ADMSCEVs therapy for PR-AKI. KEGG analysis was performed for positive-ion-features-derived chemicals significantly upregulated in PR-AKI group versus normal. We found that six metabolic pathways were significantly enriched (*P* < 0.05), including beta−alanine metabolism, galactose metabolism, fatty acid degradation, amino sugar and nucleotide sugar metabolism, histidine metabolism, and lysine degradation. A total of six chemicals were found to map on these six pathways (Fig. 4A). In parallel, KEGG analysis was performed for chemicals significantly downregulated in ADMSCEVs treatment group versus PR-AKI group. We found that five metabolic pathways were significantly enriched (*P* < 0.05), including cysteine and methionine metabolism, galactose metabolism, vitamin B6 metabolism, amino sugar and nucleotide sugar metabolism, and lysine degradation. A total of five chemicals were found to map on these five pathways (Fig. 4B). We hypothesized that chemicals satisfying the following criteria at the same time were the potential targets of ADMSCEVs therapy for PR-AKI: (1) When compared with the normal control group, the chemicals enriched in the pathways significantly upregulated in PR-AKI group; (2) when compared with PR-AKI, chemicals enriched in pathways significantly downregulated in ADMSCEVs treatment group. A total of three chemicals (carnitine, melibiose, d-Glucosamine) met the above three criteria.

Similarly, KEGG analysis was performed for negative-ion-features-derived chemicals significantly upregulated in PR-AKI group versus normal. We found that two metabolic pathways were significantly enriched (*P* < 0.05), including pyrimidine metabolism, and galactose metabolism. A total of three chemicals were found to map on these two pathways (Fig. 4C). Meanwhile, KEGG analysis was performed for chemicals significantly downregulated in ADMSCEVs treatment group versus PR-AKI group. We found that three metabolic pathways were significantly enriched, including galactose metabolism, pyrimidine metabolism, and tryptophan metabolism. A total of four chemicals were found to map on these three pathways (Fig. 4D). We used the same screening criteria as described above and revealed three negative-ion-features-derived chemicals (cytidine, dihydroorotic acid, stachyose) as potential targets of ADMSCEVs therapy for PR-AKI. Furthermore, heatmap clearly shows the distinct intensity patterns of these six candidate chemicals in normal physiological homeostasis, PR-AKI and ADMSCEVs treatment (Fig. 4E).

Conversely, we also assumed that chemicals satisfying the following conditions could also be potential targets for ADMSCEVs therapy for PR-AKI: (1) when compared with the normal control group, the chemicals enriched in the pathways significantly downregulated in PR-AKI group; (2) when compared with PR-AKI, chemicals enriched in pathways significantly upregulated in ADMSCEVs treatment group. However, we did not find any chemicals that met these conditions (Additional file 2: Fig. S2A–D).

In summary, six chemicals were identified as potential targets of ADMSCEVs therapy for PR-AKI as well as novel PR-AKI clinical indicators.

### Regression analysis of six candidate chemicals to PR-AKI clinical indicators

**Fig. 5:**
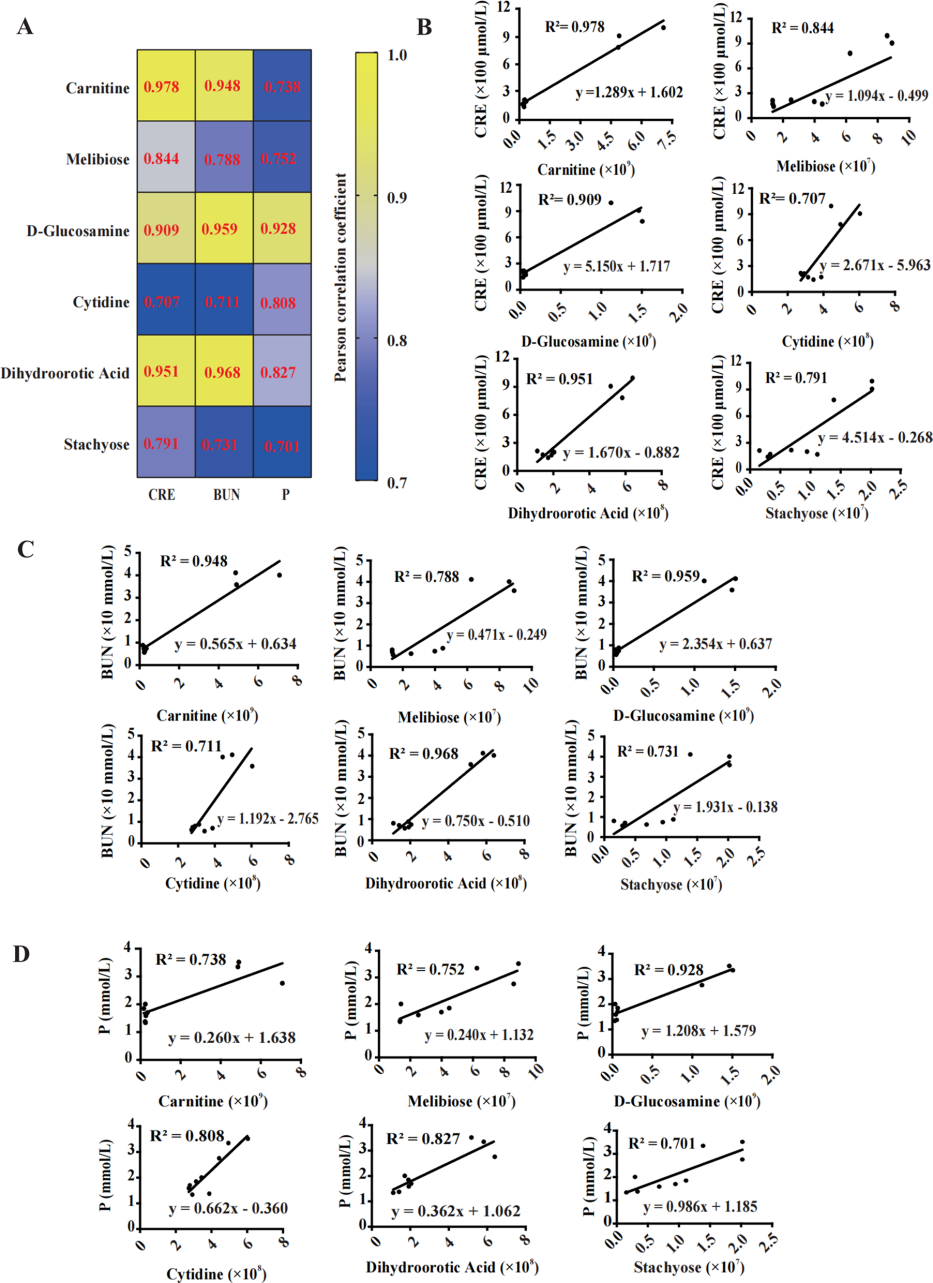
Correlation and linear regression analysis of six metabolites with clinical indicators of PR-AKI. **A** Heatmap showing the correlation coefficients between six metabolites and clinical indicators (creatinine, urea nitrogen, and plasma phosphorus) of PR-AKI. **B** Linear regression analysis of six metabolites and creatinine. **C** Linear regression analysis of six metabolites and urea nitrogen. **D** Linear regression analysis of six metabolites and plasma phosphorus. The coefficient of determination and linear regression equation are labeled for each analysis

To evaluate the indicative role of six candidate chemicals in the treatment of PR-AKI by ADMSCEVs, we performed Pearson correlation coefficient analysis for six candidate chemicals (carnitine, melibiose, d-Glucosamine, cytidine, dihydroorotic acid, stachyose) and clinical indicators of PR-AKI (creatinine, urea nitrogen, plasma phosphorus). The heatmap showed that the six candidate chemicals were strongly correlated with creatinine, urea nitrogen, and plasma phosphorus (Fig. 5A).

Furthermore, we carried out linear regression analysis between six candidate chemicals and creatinine, respectively. There was a high linear regression between the three chemicals (carnitine, d-Glucosamine, dihydroorotic acid) and creatinine (*R*^2^ > 0.9) (Fig. 5B). Interestingly, these three chemicals also showed a high linear regression with urea nitrogen (*R*^2^ > 0.9) (Fig. 5C). In contrast, only d-Glucosamine showed a high linear regression with plasma phosphorus (*R*^2^ > 0.9) (Fig. 5D).

The results above suggest that carnitine, melibiose, d-Glucosamine, cytidine, dihydroorotic acid, and stachyose can be used as complementary indicators to determine the dynamic process of PR-AKI occurrence and cure in the future.

## Discussion

**Fig. 6:**
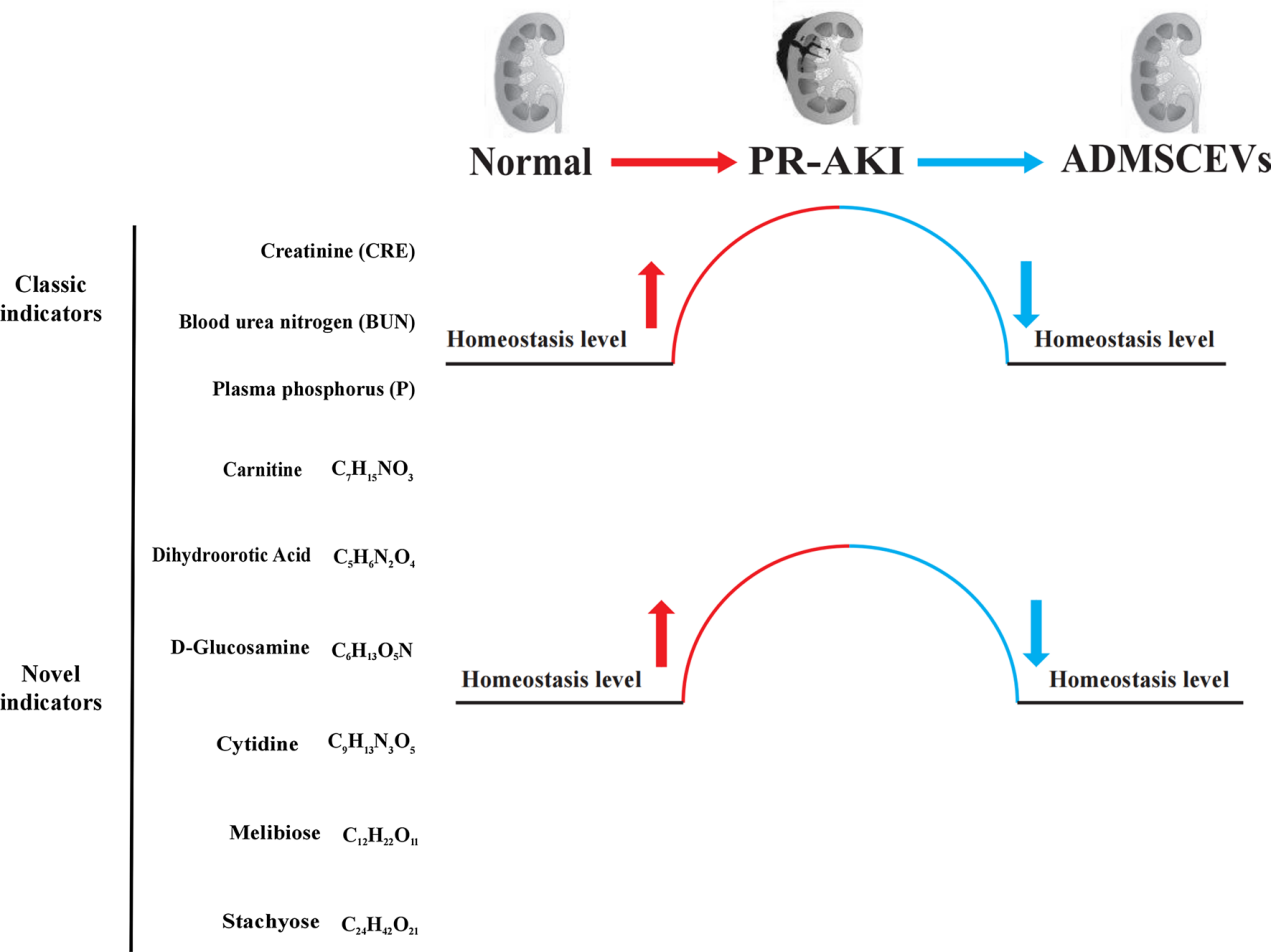
Three classic indicators and six novel indicators of PR-AKI. Changes of these indicators in cats at three states (normal, PR-AKI, and ADMSCEVs treatment) are shown

In this study, PR-AKI cat models were used to prove for the first time that ADMSCEVs can effectively promote the repair of PR-AKI. Furthermore, the plasma metabolomics study of PR-AKI cats treated with ADMSCEVs revealed that six metabolic compounds (carnitine, melibiose, d-Glucosamine, cytidine, dihydroorotic acid, stachyose) were potential targets for ADMSCEVs in the treatment of PR-AKI as well as chemical markers to indicate the dynamic process of PR-AKI occurrence and recovery (Fig. 6).

In recent years, several studies have proved that extracellular vesicles of mesenchymal stem cells (MSCs) derived from bone marrow, umbilical cord, or adipose tissue have positive therapeutic effects on renal injury [8]. Extracellular vesicles can repair renal cells, alleviate kidney injury and promote the recovery of physiological function of the kidney. We speculated that the therapeutic effect of ADMSCEVs on PR-AKI may follow a similar mechanism above. The underlying biological mechanisms include inhibition of renal apoptosis and fibrosis, reduction in renal inflammation, promotion of renal proliferation and angiogenesis [9–11]. Extracellular vesicles are rich in mRNAs, microRNAs and proteins with regulatory functions. For example, miR-29 and miR-24 in MSCs-derived extracellular vesicles have been reported to inhibit apoptosis [12–14]. Other miRNAs, including miR-16, miR-15a and miR-15b in MSCs-derived extracellular vesicles, can alleviate renal inflammation and inhibit renal fibrosis by suppressing the expression of CX3CL1 [15]. In addition to miRNAs, mRNAs in MSCs-derived extracellular vesicles, such as IGFR1 mRNA, can enhance proliferation and repair of renal tubular epithelial cells [16]. MSCs-derived extracellular vesicles are also rich in proteins with various biological activities, including angiogenesis (vascular endothelial growth factor, angiopoietin related protein 4), apoptosis (netrin-1), inflammatory response (tumor necrosis factor-inducible gene 6 protein) and extracellular matrix remodeling (matrix metalloproteinase-19, transforming growth factor beta 1) [17, 18]. We speculate that the above miRNAs, mRNAs and proteins may also exist in ADMSCEVs, and it is the comprehensive effect of these components that make ADMSCEVs significantly promote the recovery of PR-AKI cats. Specific mechanisms of ADMSCEVs promoting the repair of PR-AKI need to be further studied.

The regulatory effect of ADMSCEVs is finally reflected in the changes of metabolites in the metabolic process. In this study, the changes of plasma metabolites in PR-AKI cats treated with ADMSCEVs were analyzed by UHPLC-MS/MS. Among them, six metabolites were found to be potential targets for ADMSCEVs treating PR-AKI, and to be novel markers of PR-AKI.

Carnitine is a quaternary ammonium compound. The main physiological function of carnitine is to transfer long-chain fatty acids to mitochondria for oxidation, thereby producing energy. In addition, carnitine is involved in removing metabolites from cells [19]. At the organ level, kidney is the main regulator of plasma carnitine homeostasis. It has been reported that carnitine has a protective effect on AKI caused by ischemia–reperfusion [20]. In this study, the level of carnitine in PR-AKI cats increased sharply, which may be caused by the disturbance of carnitine homeostasis. The rise of carnitine in cats with PR-AKI may also be a self-protective mechanism. In addition, highly consistent linear regression between carnitine and creatinine/urea nitrogen suggests that carnitine can be used as a new marker for PR-AKI in the future.

d-Glucosamine is a monosaccharide that contains an amine group in place of one of the hydroxyl groups. Glucosamine is an effective drug in the treatment of osteoarthritis [21]. Glucosamine can cause apoptosis in kidney tubular and mesangial cells as well as overexpression of transforming growth factor β1 (TGF-β1) and connective-tissue growth factor (CTGF), which are potent inducers of mesangial and interstitial tubulointerstitial fibrosis [22]. Therefore, we speculate that glucosamine may be one of the main agents causing PR-AKI in this study.

Dihydroorotic acid and cytidine are two substances in pyrimidine metabolism, in which dihydroorotic acid is mainly involved in nucleotide anabolism, while cytidine is produced by nucleotide catabolism. Orotic acid is a downstream metabolite of dihydroorotic acid. When the urea cycle is interrupted, orotic acid synthesis is supposed to increase. The synthesis of orotic acid is initiated by the formation of carbamyl phosphate in the cytoplasm, where the ammonia comes from glutamine. In mammals, carbamyl phosphate is formed in the first step of mitochondrial urea synthesis in the liver. In PR-AKI, the body's ability to detoxify ammonia is insufficient, resulting in the accumulation of urea nitrogen. Carbamyl phosphate leaves the mitochondrial urea synthesis pathway and enters the pyrimidine pathway to stimulate the biosynthesis of orotic acid, which may lead to the rise of dihydroorotic acid [23]. Correlation analysis between dihydroorotic acid and urea nitrogen also supports this speculation. In PR-AKI, there is a lot of cell damage, so that a lot of nucleotides need to be broken down, which may be responsible for the rise of cytidine.

Melibiose and stachyose are the products of galactose metabolism, and stachyose is the precursor of melibiose. Melibiose has been shown to effectively inhibit oxidative stress and the upregulation of inflammatory factors in mice [24, 25], while stachyose has also been shown to inhibit inflammation [26, 27]. In the case of PR-AKI, the upregulation of melibiose and stachyose may be a spontaneous protective mechanism.

We are also aware of the limitations of this study. First, the exact mechanism behind the efficacy of ADMSCEVs on PR-AKI needs to be further clarified; Second, the validity of the six metabolites obtained by screening a small number of artificial PR-AKI cat models needs to be further verified in a large number of clinical feline PR-AKI cases; Finally, prognostic outcomes need to be tracked to assess the long-term benefits of ADMSCEVs in the treatment of PR-AKI.

## Conclusions

In conclusion, we have demonstrated the efficacy of ADMSCEVs in the treatment of PR-AKI, and revealed six metabolites to be potential targets for ADMSCEVs therapy as well as novel PR-AKI markers. The findings of this study will provide new clues to improve the clinical treatment of both animal and human PR-AKI patients with ADMSCEVs.

## Supplementary Information

**Additional file 1. Table S1**: Results of metabolomic analysis.

**Additional file 2. Fig. S1**: Dynamic changes of leukocytes, plasma phosphorus and plasma calcium level. **Fig. S2**: KEGG analysis of plasma metabolites.

## Data Availability

Not applicable.

## Abbreviations

AKI: Acute kidney injury
PR-AKI: Postrenal acute kidney injury
ADMSCEVs: Adipose mesenchymal stem cell-derived extracellular vesicles
ADMSCs: Adipose mesenchymal stem cells
MSCs: Mesenchymal stem cell-derived
AUC: Artificial urinary closure
UHPLC-MS/MS: Ultra-high-performance liquid chromatography-MS/MS
TGF-β1: Transforming growth factor β1
CTGF: Connective-tissue growth factor
ESI: Electrospray ionization
QC: Quality control
PCA: Principal component analysis
