# Supplementary material for: Rapid recovery of male cats with postrenal acute kidney injury by treating with allogeneic adipose mesenchymal stem cell-derived extracellular vesicles
Source: Stem Cell Res Ther. 2022 Jul 28;13:379. doi: 10.1186/s13287-022-03039-z (PMC9331582; doi:10.1186/s13287-022-03039-z)
Supplement: Supplementary file 2 — Additional file 2. Fig. S1: Dynamic changes of leukocytes, plasma phosphorus and plasma calcium level. Fig. S2: KEGG analysis of plasma metabolites. [file 13287_2022_3039_MOESM2_ESM.docx]

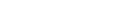

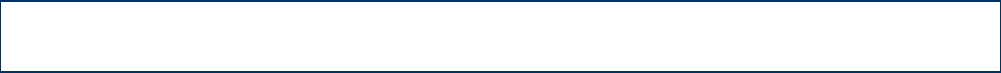


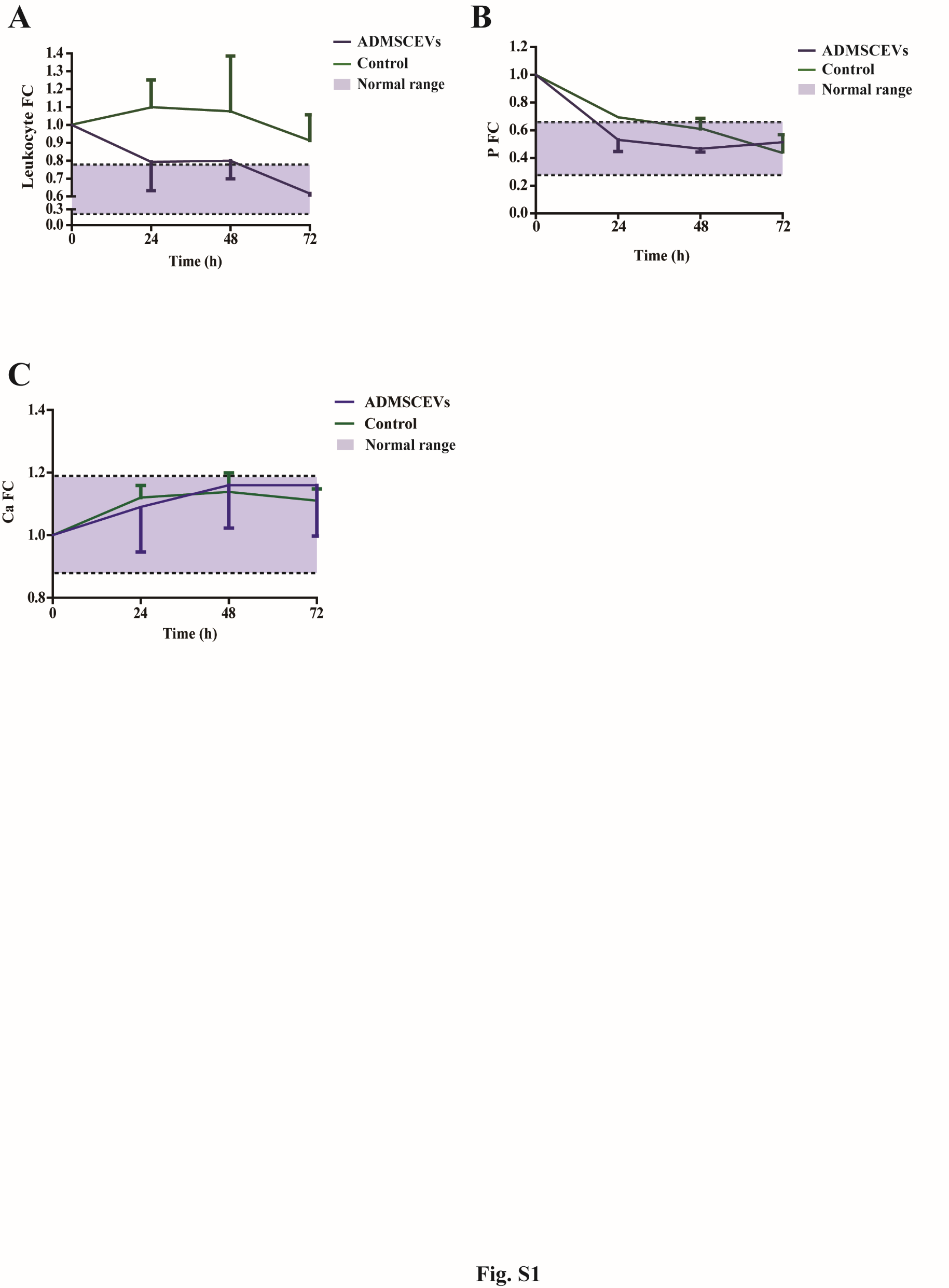


Fig. S1 Dynamic changes of leukocytes, plasma phosphorus, and plasma calcium level. A. Dynamic fold changes in Leukocytes of PR-AKI cats treated with control infusion and ADMSCEVs. B. Dynamic fold changes in plasma phosphorus level of PR-AKI cats treated with control infusion and ADMSCEVs. C. Dynamic fold changes in plasma calcium level of PR-AKI cats treated with control infusion and ADMSCEVs.


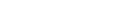

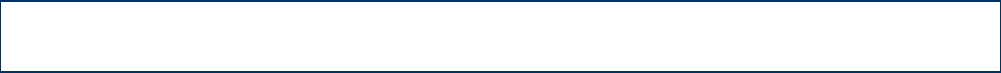


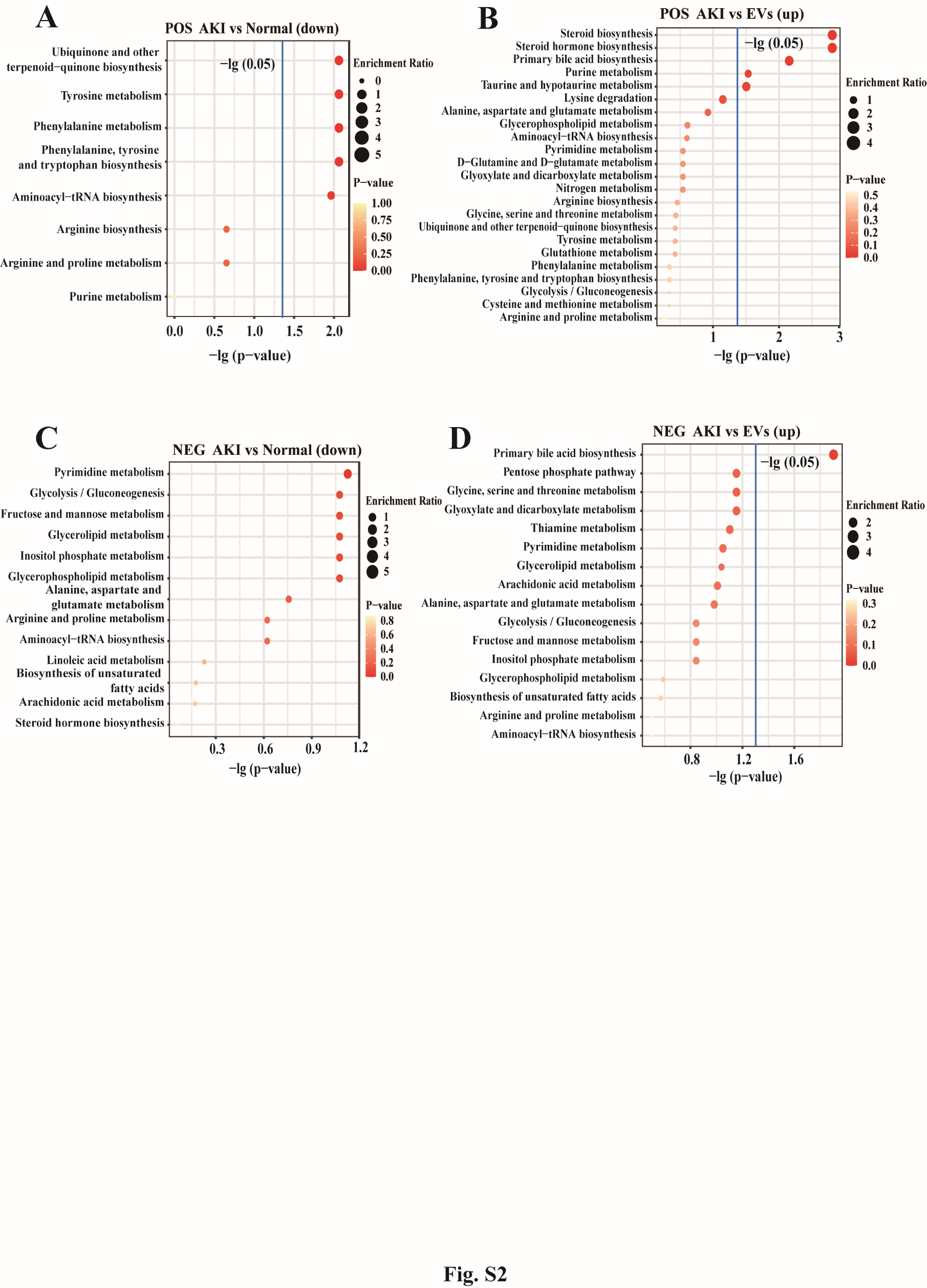


Fig. S2 KEGG pathway analysis of cat plasma metabolites. A. KEGG pathway analysis of significantly down-regulated metabolites (annotated from positive ion features) in normal cats compared with PR-AKI cats. B. KEGG pathway analysis of significantly up-regulated metabolites (annotated from positive ion features) in ADMSCEVs-treated cats compared with PR-AKI cats. C. KEGG pathway analysis of significantly down-regulated metabolites (annotated from negative ion features) in normal treatment group compared with PR-AKI group. D. KEGG pathway analysis of significantly up-regulated metabolites (annotated from negative ion features) in ADMSCEVs-treated cats compared with PR-AKI cats. The blue line indicates −lg (p−value = 0.05).
